# A Polish Pilot Programme of Coordinated Care: A Herald of Change or a Missed Opportunity? A Critical Debate

**DOI:** 10.3389/fpubh.2020.00360

**Published:** 2020-08-04

**Authors:** Monika Karasiewicz, Ewelina M. Chawłowska, Agnieszka Lipiak, Rafał Staszewski

**Affiliations:** ^1^Laboratory of International Health, Department of Preventive Medicine, Poznan University of Medical Sciences, Poznań, Poland; ^2^Department Hypertensiology, Angiology and Internal Diseases, Poznan University of Medical Sciences, Poznań, Poland

**Keywords:** healthcare reform, access to care, primary care, integrated care, coordinated care

## Abstract

The part of the health system which is essential for achieving universal health coverage is primary health care. Recognising the need to reform the health system and primary care in particular, on 1 July 2018 the Polish government launched POZ Plus—a pilot programme of coordinated primary care. Its objectives are to improve the quality and coordination of care and to expand its scope. The objectives are to be delivered through preventive health checks in patients aged 20–65 years, predefined chronic disease management programmes, as well as coordination and monitoring of care carried out by primary care teams. The programme provoked a heated debate and mixed reactions from different stakeholders in Poland. On the positive side, POZ Plus improves patient experience and seems to be a promising preventive tool. During the first 14 months of programme life, 10,956 health checks resulted in 13,361 new diagnoses. The critics of the programme point out that the scope of care is too wide and unnecessary in general population, and the health checks are too long, given the scarcity of medical professionals in Poland. The programme requires significant up-front investment of time and resources, thus favouring big clinics from densely populated areas. Financing may be sufficient during the pilot phase, but the programme may turn out to be too costly for country-wide implementation. The programme is a promising start toward achieving better primary care coordination in Poland. However, its success is conditional on sound public financing, rational workforce strategy, and close collaboration of all stakeholders.

## Introduction

Celebrations of the World Health Day marking the 70th anniversary of the World Health Organisation once again turned our attention to the Health for All (HFA) policy which, aiming at universal health coverage, is WHO's main mission. According to the Declaration of Alma-Ata of 1978 (1), the key system that can lead to the implementation of HFA is primary health care (PHC) with its comprehensive approach to health. The Declaration states that “governments should formulate national policies, strategies, and plans of action to launch and sustain primary health care as part of a comprehensive national health system and in coordination with other sectors.” This vision was further developed in the WHO global strategy on people-centred and integrated health services adopted in 2015, promoting health care that is “integrated around people's needs and effectively coordinated across different providers and settings” (2). Over the years, a whole family of concepts related to care integration (integrated care, coordinated care, managed care, seamless care, etc.) has been developed. In an attempt to systematise them, Valentijn et al. (3) constructed a comprehensive conceptual framework for integrated care based on the integrative functions of primary care. The framework lists the following dimensions of integration: clinical (coordination of person-focused care into a single process across time, place, and discipline, such as through the use of shared protocols), professional (coordination of services across various disciplines, such as through teams of multidisciplinary professionals), organisational (coordination of the efforts of different organisations aiming to deliver comprehensive care), functional (coordination of support functions, such as information or financial systems, between organisations and professionals), normative (use of shared mission and values across the system), and finally system coordination (incorporation of all the above levels, leading to the alignment of rules and policies within a system) (3, 4). Ideally, achieving such multi-level integration of care might be a solution to the growing problems of chronic and non-communicable diseases (NCDs) as well as multimorbidity (5).

Although the Polish health care also faces these problems, the integrated care ideas may seem unattainable to Polish patients, healthcare providers, and control institutions alike, who rank the health system as a whole, and PHC in particular, very low (6–11). Given the alarming NCD trends reported for Poland (12–14), this is the right moment to try to achieve better health of citizens through PHC. To this end, several solutions have been introduced lately by the Polish government. The aim of this paper is to describe and analyse one of these solutions: the POZ Plus pilot programme of coordinated primary care. Moreover, we are going to present the debate on POZ Plus among its stakeholders in Poland, as well as our views on the feasibility of implementing the programme on a wider scale. This way, we would like to join the international debate on coordinated care within PHC and within the healthcare system as a whole.

## The Need for Reforms

The Polish health care has changed considerably within the last three decades in terms of quality, financing, and management. Before 1989, when Poland was part of the communist bloc, the healthcare system was modelled on the Soviet pattern (the so-called Semashko model) of strictly regulated, centralised, and state-funded care. The 1990s saw a gradual transition to a hybrid, partially decentralised system with a mixed nature and a bigger role assigned to local self-governments and non-public providers and with mandatory health insurance. At present, the health insurance funds are managed by NFZ (Narodowy Fundusz Zdrowia—National Health Fund)—a monopsonist and the sole purchaser of public health services. The health insurance, which is in fact a dedicated tax paid mainly on income from work and pensions, comprises 87% of public health financing. The remaining 13% comes from general taxes and finances emergency care, some highly specialised procedures, as well as insurance contributions for those who are either unable to pay or are exempt from paying them (e.g., uninsured children, the unemployed who do not receive unemployment benefits, small farmers). Although health protection is a constitutional right in Poland, full access to public health care is available to the insured only. At the end of 2017, 91% of Poles were insured and thus entitled to use the public system (7).

Yet despite the long-standing tradition of publicly financed health care, the Polish system has had serious difficulties for years. It is confirmed in a number of studies, including the Euro Health Consumer Index reports of recent years, in which Poland has been consistently scoring very poorly among 35 European countries: it ranked 31st in 2016, 29th in 2017, and 32nd in 2018 (8–10). The reasons include insufficient transparency, clarity, and accountability, limited access to diagnostic and treatment options, poor cancer survival rates, a big proportion of private spending on health care in the face of queues to public providers, difficulty accessing prevention, and screening measures in PHC (7, 15–17), low public health expenditure (4.6% of gross domestic product in 2017) and, last but not least, a small number of practising doctors (2.4 per 1,000 population in 2016 compared to the European Union average of 3.6) and nurses (5.2 per 1,000 population in 2016 compared to the EU average of 8.4) (7). The dramatic personnel shortages are caused by the lack of a comprehensive government workforce planning strategy in the face of health professionals' low interest in taking up work in Polish health care and their emigration resulting from poor working conditions, low salaries, barriers to professional development, and very heavy workload aggravated by shortages of support staff (7).

Although PHC is an entry point to the public system, it is also one of its weakest links. Historically, during the communist era, it was regarded as inferior to inpatient care with its extensive infrastructure and high prestige of specialist physicians (8). As a result, it may be concluded from the available reports that PHC is now not only underfinanced, but also understaffed. Only 9% of all practising physicians, compared to the EU average of 23%, work in primary care (7). Nurses are also scarce; their education is focused on inpatient care (18, 19) and their role in PHC is often marginalised. Finally, the funding is far too low given the fundamental role of PHC. In 2017, PHC provided 52.9% of all outpatient consultations, but received only 13.4% of public health spending (20). It is little wonder then that one of the most important roles of primary care, i.e., prevention, appears to be seriously compromised. It is corroborated by the Polish Supreme Chamber of Control, which found that Polish PHC fails to systematically provide and document preventive care, including basic diagnostic tests and procedures. The Chamber's 2015 audit of PHC records showed that among patients with high risks of developing NCDs, weight and body mass index measurements were documented in only 30.7% of the records, blood pressure in 66.5%, and blood glucose in 44.3% (12). Furthermore, the perceived quality of ambulatory care in Polish PHC as measured by doctors spending enough time with patients in a consultation, doctors involving patients in decisions about care and treatment, and the general quality of PHC service is reported to be among the lowest in the EU (6).

## The Design of the Pilot Programme

Faced with this difficult situation, in autumn 2017 the Polish parliament passed the Primary Health Care Act (21), which outlines a plan of gradual reforms of the Polish primary health care to be implemented fully by 1 January 2025. The new legislation provides for a progressive increase of health expenditure from government funds, and PHC is to receive the increased spending first. The Act also contains provisions changing the organisation and financing of care, increasing the role of health education, and prevention in PHC, and improving the quality of care. The implementation of these reforms is preceded by a pilot programme of “coordinated care organisation” called POZ Plus (PHC Plus) launched at selected healthcare facilities on 1 July 2018 and planned to end on 31 December 2021.

The programme, worth nearly PLN 76 million (almost USD 20 million) (22), is coordinated by NFZ in collaboration with the World Bank as part of the operational programme Knowledge Education Development 2014–2020, which is financed from the European Social Fund, and cofinanced from NFZ budged (23). According to the relevant legislation (23, 24), the main objectives of the programme are: (1) to improve the quality of PHC; (2) to increase the number of healthcare services available in PHC; and (3) to improve the coordination of PHC. The model is to be patient-centred and preventive rather than service-centred and remedial. It was developed in the process of analysing and reviewing coordinated care examples from other countries (North West London Integrated Care, Alaskan Nuka System of Care, Kaiser Permanente from the USA, German Gesundes Kinzigtal, Program-for-Results reforms from China and Costa Rica, and many more) (7, 22, 25, 26). It was also consulted with a number of stakeholders: Ministry of Health, NFZ, healthcare providers, non-governmental organisations, healthcare professionals (26) and, last but not least, PHC patients (*n* = 1,024) (27). Finally, in 2017 a small-scale test of coordinated PHC was carried out on 122 patients of 13 PHC clinics and analysed before the POZ Plus was officially launched (28).

Out of the nearly 7,000 PHC providers operating in Poland, 874 expressed their interest in the programme, but due to limited funds only 45 clinics, distributed evenly throughout Poland, were selected. Their managers and medical staff were trained in various areas related to programme implementation (29–31). Since 1 July 2018, the model has been implemented by 42 clinics providing primary care to ~300,000 patients (23, 32).

**Figure 1:**
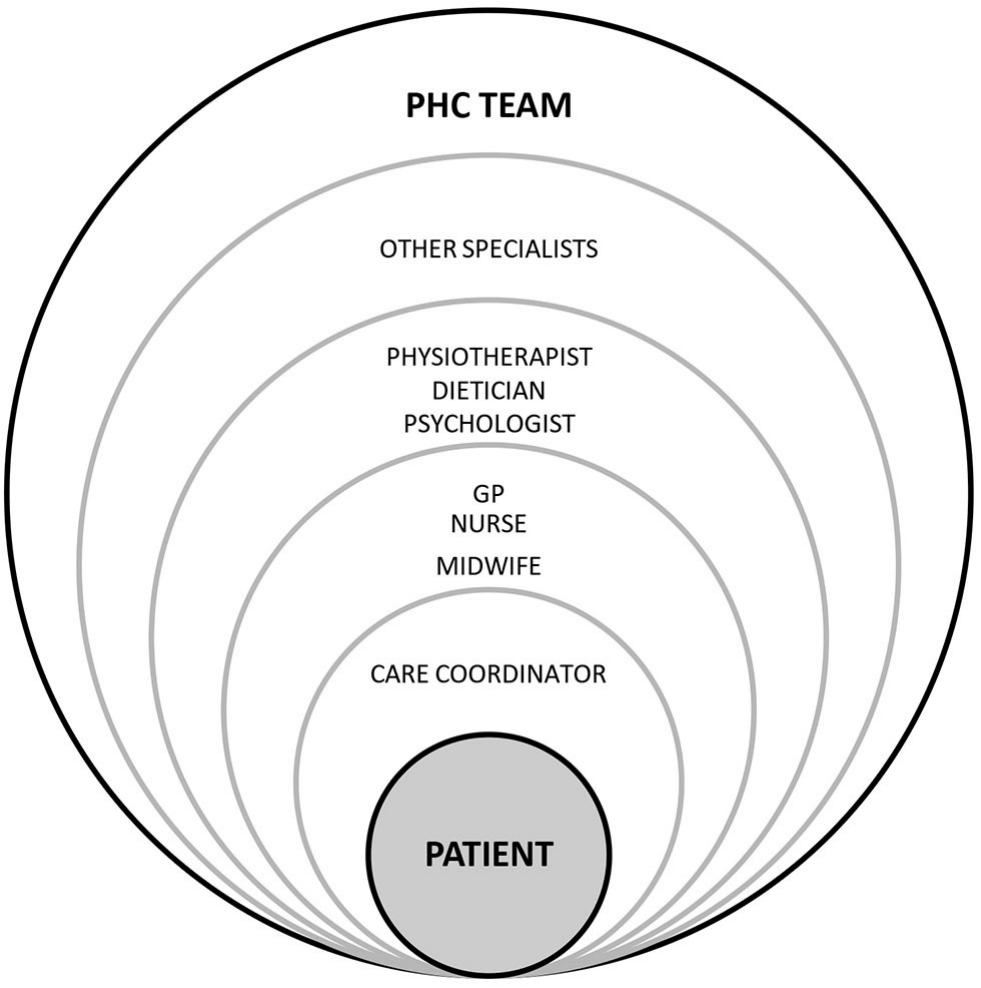
POZ Plus: the model of patient care.

The model is based on building a PHC team whose core members are a GP (general practitioner), nurse, midwife, and care coordinator. They closely collaborate with specialists: a physiotherapist, dietician, psychologist, diabetologist, endocrinologist, cardiologist, neurologist, pulmonologist, orthopaedist, and physiotherapist (33, 34). The GP is a decision-maker as regards necessary interventions, a nurse gains more responsibilities in disease prevention and education, and a care coordinator is responsible for administrative work, scheduling diagnostics and consultations, inviting patients to examinations, and coordinating communication between patients and healthcare professionals [(23, 33, 35) see Figure 1].

The scope of care is wider than that normally offered by PHC clinics. The core of the preventive part of the programme—a comprehensive basic health check—is to be offered every 5 years to adult patients aged from 20 to 65 years. The minimum number of health checks to be conducted by all the participating clinics is 41,878. The number per clinic is proportionate to the number of its registered patients (23, 32). It should be stressed that the health checks are aimed at the patients whose health status is partly or completely unknown to a clinic. That is why the checks may only be offered to the patients who have not undergone any diagnostic procedures being part of the checks and have not been treated for a chronic illness within any part of the public healthcare system within the previous 12 months.

The basic check includes history taking, physical examination, basic measurements, and laboratory diagnostics. If any risk factors or diseases are identified, an in-depth check follows with additional diagnostic tests selected from a pre-defined catalogue depending on clinical indications as well as a patient's age (34). Based on check and diagnostic test results, a patient is assigned one of the four health statuses: healthy with no risk factors, healthy (no symptoms) with risk factors, chronically ill but stable (no current symptoms), or chronically ill and in need of stabilising (with current symptoms). This patient stratification process is meant to streamline the number and kind of services provided to specific patient groups (23, 34). Each “stratified” patient is offered a personalised care plan (PCP), which he/she can actively discuss with a doctor. The PCP includes check results and medical recommendations, and constitutes a basis for further interventions, e.g., educational visits or consultations with specialist physicians. A patient cannot choose specialists freely but may only consult the specialists contracted to collaborate with his/her PHC clinic as part of POZ Plus.

**Figure 2:**
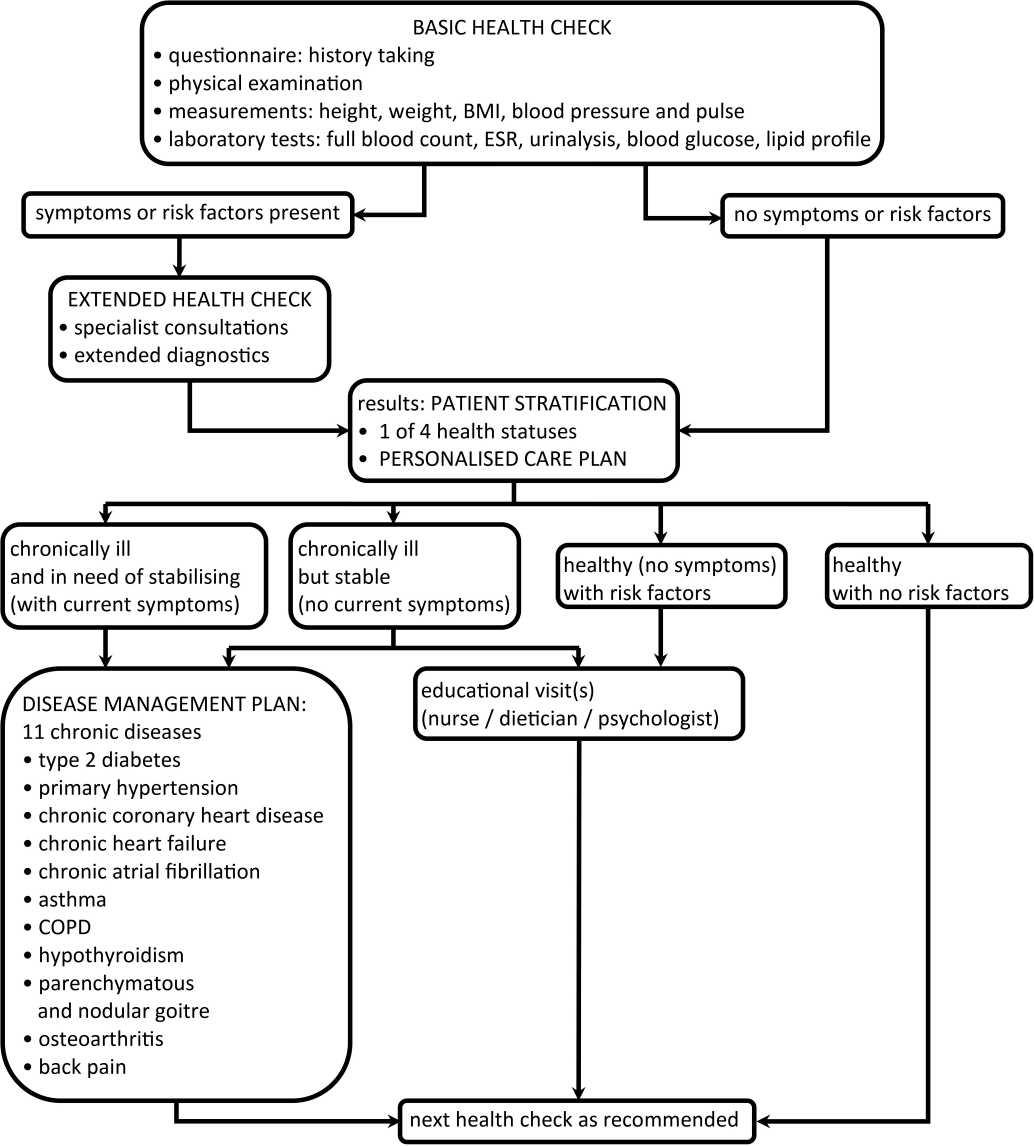
POZ Plus: tasks and activities.

After stratification, a patient categorised as healthy is invited to undergo the next check in 3 years or more (except when in urgent situations such as infections or other acute states). A patient categorised as ill may qualify to take part in a disease management programme (DMP), which follows pre-defined diagnostics and treatment pathways (23, 34). DMPs are designed to facilitate and accelerate diagnosis and treatment of 11 chronic diseases found to be the most prevalent in Polish PHC—patients diagnosed with these conditions constitute 54.2% of all primary care patients in Poland, and the related consultations account for 24% of all primary care services [(36) see Figure 2].

The quality and efficiency of the model are to be monitored by means of periodic patient satisfaction surveys (with Patient Activation Measure, Patient Reported Outcome Measure and Patient Reported Experience Measure as tools) and numerous other indices such as proportions of patients who are hospitalised or who seek specialist consultations outside the programme (22, 37). Basic country-wide programme statistics are compiled, periodically updated, and presented on the dedicated NFZ website akademia.nfz.gov.pl (38).

## The Current Debate on the Pilot Programme

### Preliminary Results

According to the NFZ statistics, the 10,956 health checks carried out from 1 July 2018 to 31 August 2019 (39) resulted in as many as 13,361 diagnoses, with the most frequent being cardiovascular diseases (41%), followed by rheumatic, and neurological diseases (28%), endocrinological diseases (15%), diabetes (11%), and pulmonary diseases (5%) (40). Similar findings came also from the small-scale test of the model conducted before POZ Plus was launched. The health checks performed on just 21 days in a group of 122 patients resulted in numerous diagnoses of not only diseases but also risk factors, [for e.g., 19 cases of increased alcohol addiction risk and 12 cases of increased depression risk (28)]. The researchers who analysed these results concluded that the model seems to be a useful preventive measure feasible for PHC clinics with at least basic health care coordination, but needs further investigation in clinics with different levels of health care coordination (28).

### Scope of Care

The readily available extended diagnostics, easy access to specialists, as well as scrupulous attention and considerably longer time devoted to patients within POZ Plus are likely to meet with positive patients' reactions, since these are the elements lacking from most patient-doctor interactions in Polish PHC. There are, however, critical voices saying that the scope of examinations and specialist diagnostics available to patients is too wide. The number of basic health checks per clinic is said to be excessive and based on an unrealistic assumption that a PHC team will be able to perform, on average, two such lengthy (~60 min long) consultations a day (41, 42). It is also raised that referring patients to some complicated procedures available in the programme (e.g., plethysmography or computed tomography angiography) may go beyond the competencies of PHC doctors. There is criticism of using universal health checks in generally healthy patients (41, 42), especially as there is mixed evidence of their positive influence on morbidity and mortality (43–47). Systematic assessment of coronary risk by means of laboratory tests in 20- to 35-year-olds seems to many experts neither evidence-based nor adequate for patients' needs (41, 42, 48, 49). NFZ addresses the criticism, stressing that the health checks are performed in patients whose health status is unknown to their PHC providers (50), and in-depth checks—only in patients with risk factors. Thus, the examinations serve as a secondary prevention measure and a basis for patient stratification, which, coupled with e-health solutions, is expected to lower the number of avoidable patient visits (50). Some PHC managers claim that the new solutions have indeed caused a 30% drop in the number of visits (19). NFZ also declares that the diagnostics and treatment pathways are based on expert literature and guidelines from national and international bodies (e.g., European Society of Cardiology or Polish Psychiatric Association) (50).

### Workforce

The view emerging from our discussions with frontline POZ Plus staff and our own observations is that the new kind of work organisation within POZ Plus is both an opportunity and a challenge. It can foster team spirit and professional collaboration. The health professionals involved in the programme can share their experiences using a dedicated official e-platform called Akademia NFZ, as well as closed unofficial support groups on popular social media. Such solutions definitely help to share ideas and integrate PHC providers. For physicians, the programme is an opportunity, unique in the Polish public healthcare system, to provide their patients with diagnostics and treatment in accordance with the doctors' best knowledge and experience in primary care without the need to constantly worry about the costs. Generalists can finally go beyond giving prescriptions and referrals and are able to devote their time and the available means to give their patients well-coordinated care. According to experts, the programme also means the empowerment and strengthening of nurses' position in PHC (18, 51). In our view, the new role of care coordinators saves doctors a lot of administrative chores and is a chance to redress the problem of GP shortages. Unfortunately, the programme did not include any training for coordinator candidates, which may result in difficulties recruiting people with the necessary competencies: good organisation and communication skills, team spirit, and digital literacy. A possible solution could be to make full use of support staff such as public health specialists, a few 100 of whom graduate from Polish medical universities each year.

### Inequalities

On a closer look at the infrastructure and functioning of the participating providers (52), it turns out that they are among the best managed PHC clinics in Poland, exceptionally well-prepared in terms of equipment, facilities, new technologies, staffing, and enthusiasm. Perhaps that explains why there were no small practices among the initially selected 45 providers (53), and why 3 more clinics quit later on in the course of the programme. According to providers, POZ Plus appears to favour mostly big clinics from densely populated areas because the entry conditions are unattainable for small clinics due to insufficient human resources, lack of workspace for the newly employed coordinators, and extra workload arising from lengthy health checks, complicated reporting, and unwieldy dedicated IT tools incompatible with other software used in health care (19, 41, 42, 54). That is why it is feared that the programme will lead to increased inequalities between PHC providers by strengthening the already strong ones and not providing enough support to the weaker ones.

### Financing

The course of the programme to date seems to indicate the need to adjust its financing in terms of both scheme and amount. It appears reasonable to adjust the basic programme financing scheme—capitation—to meet the needs of rural PHC clinics with dispersed, remote, or difficult-to-reach populations and thus make a step toward eliminating health inequalities mentioned above. As regards the amount, it can only be hoped that the government will keep its promises and gradually increase health expenditure. Although universal implementation of coordinated care could lead to more rational spending, some experts, and providers fear that the programme is unworkable as a long-term approach. While its pilot stage receives sufficient funding, it will be unavailable as soon as the EU funds are consumed, which makes country-wide implementation doubtful (54, 55).

### Care Coordination

One might wonder if POZ Plus does what it declares to do. Does it provide coordinated care, as the name of the programme suggests? Given the preliminary programme result statistics, we believe POZ Plus appears to be working toward this aim. The model increases collaboration within PHC and gives each team member an important role. If we refer to the wider framework of integrated care provided by Valentijn et al. (3), we can see that the programme is basically an effort to replace care provided by individual professionals with the work of multidisciplinary teams united in a single standardised patient-focused care process. Therefore, the primary focus of the programme is professional and clinical integration. As for organisational integration across different stakeholders, it is limited basically to health care. We feel that the pilot would greatly benefit from multi-sectoral collaboration, especially with an aim to address complex health needs of deprived populations. In various coordinated care models around the world, it has often been achieved through collaboration between health care and social care (56, 57). Furthermore, POZ Plus also entails a degree of functional integration in the form of selected support functions (a uniform IT system, management model, and reporting system), whose use is simply required by the government as the programme initiator and leader. Also normative integration with respect to the programme mission applies to a certain extent, although not everyone shares the same vision of care coordination. A wide interest in the programme on the part of PHC providers showed that they share the vision of patient-centred care, recognise the need to reform PHC and look for opportunities for implementing change. The government attempted to develop a shared vision through wide consultations before programme launch (26). The currently operating formal and informal platforms for the exchange of experiences and ideas between stakeholders are conducive to further integration of programme mission. At the same time, there are opinions among providers that the government did not treat seriously enough the criticism and concerns voiced by non-governmental stakeholders (41, 42, 48), which is why they proposed alternative coordinated care models (58). In effect, it would be unfeasible to expect full system integration from such a small and widely debated pilot programme. The limited scale of the pilot programme, which is implemented by only 42 PHC clinics of out 6,711 providers operating in 2018 (59), raises doubts if its results will be transferable to country level or clear enough to contribute to the discussion on the future directions of Polish health policy (60). Given all the above, it seems that Poland has only just started on the way toward more coordinated care and the programme involves only some of the levels of integration mentioned before.

## Discussion

The Polish healthcare system badly needs care integration in order to eliminate care gaps, queues to specialists, bottlenecks, and unnecessary duplicate spending. Obviously, this cannot be achieved through coordinated PHC alone. As it was often stressed in literature (8, 16, 59, 61), it requires wider structural reforms, a comprehensive look at the entire healthcare system, and a unified strategic vision.

In our opinion, if Polish PHC is to gain more prominence and POZ Plus is to be implemented on a wider scale, the three issues which need to be addressed urgently are healthcare funding, workforce strategy, and collaboration between the government, and other stakeholders.

As regards the funding, it needs to be both substantially increased and rationally allocated. One option could be gradual reallocation of funds in the healthcare system in such a way as to reduce the overreliance on hospital care in favour of PHC, which is proposed all over the world (6, 9–11). In the long run, however, Poland will have to follow the lead of other developed countries and spend more on the entire health system. We fear that without sound financial background Poland may not be able to provide coordinated PHC to all its citizens. The gradual increase of public spending on health care provided for in Polish legislation is expressed as a percentage of gross domestic product. As GDP is expected to shrink due to the 2020 COVID-19 pandemic and the resulting global crisis, it is difficult to predict if the government will be able to stick to its plans of raising health expenditure.

Secondly, in our view a rational long-time workforce strategy in health care must be implemented. Again, it should consist not only in attracting new workforce by means of financial incentives, but also in allocating the available staff and workload strategically. Possible solutions could include reducing bureaucratic burdens (for e.g., through elimination of duplicate reporting and better integration of IT systems) and making full use of support staff, i.e., care coordinators, who are supposed to take over most of the administrative duties. Such an important role definitely requires proper training, which is currently missing from POZ Plus and should be provided. Luckily, there are specialists in Poland who would require relatively little training: public health graduates. A few 100 people graduate in public health annually, but their potential is not fully utilised because the Polish healthcare system still lacks positions dedicated to them. In our view, they could become valuable members of PHC teams as care coordinators. However, it should be kept in mind that structural changes are also necessary to attract and keep other staff members in the Polish system: without a sufficient number of generalists, specialists, and nurses, coordinated care will not be possible.

Finally, to maximise the benefits of the PHC reform, efforts should be made to ensure good collaboration between the government and other stakeholders. As with any major reform, its success relies on commitment and close collaboration of all the stakeholders involved: the whole civil society. That is why it is crucial to listen carefully to all the concerns voiced, to discuss differences, and to try and win the stakeholders' enthusiasm. With silo thinking, hierarchical nature, and fragmentation of care deeply entrenched in Polish health care, it can be expected that the universal adoption of such a team work concept will be a painstaking process. All too often in the past the Semashko legacy came to the fore, the government stance was imposed, and public consultations were treated as an unnecessary formality. Thus, it is essential that the course of the programme should be systematically monitored, analysed, and adjusted in order to develop best possible solutions, as promised by the officials in charge (50).

Unquestionably, achieving universal health requires not only a holistic approach to patients, but also solving long-neglected ills of the healthcare system. Thus, it seems doubtful whether the coordinated PHC model will be implemented as a rule soon after the pilot programme ends. The final conclusions can be formulated only after the programme is wound up and duly evaluated, i.e., not earlier than in December 2021. However, POZ Plus has already initiated a heated discussion and offered a chance to look at the issue of coordinated care in primary settings from many different perspectives.

## Data Availability Statement

The original contributions presented in the study are included in the article/supplementary material, further inquiries can be directed to the corresponding author.

## Author Contributions

All authors made substantial contributions to the conception and design of this paper. MK and EC conceived its idea. MK and AL wrote the core of the manuscript. EC coordinated its further development. AL and RS contributed to the discussion and developed the figures. All authors read, revised, and approved the final manuscript.

## Conflict of Interest

The authors declare that the research was conducted in the absence of any commercial or financial relationships that could be construed as a potential conflict of interest.

## Abbreviations

DMP: disease management programme
EU: European Union
GP: general practitioner
HFA: Health for All
NCDs: non-communicable diseases
NFZ: National Health Fund (Polish: Narodowy Fundusz Zdrowia)
PCP: personalised care plan
PHC: primary health care
WHO: World Health Organisation.
